# Alteration of functional connectivity in autism spectrum disorder: effect of age and anatomical distance

**DOI:** 10.1038/srep26527

**Published:** 2016-05-19

**Authors:** Zhiliang Long, Xujun Duan, Dante Mantini, Huafu Chen

**Affiliations:** 1 Center for Information in BioMedicine, Key laboratory for Neuroinformation of Ministry of Education, School of Life Science and Technology, University of Electronic Science and Technology of China, Chengdu 610054, China; 2 Neural Control of Movement Laboratory, ETH Zurich, Switzerland; 3 Department of Experimental Psychology, University of Oxford, Oxford, United Kingdom; 4 Laboratory of Movement of Control and Neuroplasticity, KU Leuven, Leuven, Belgium

## Abstract

Autism spectrum disorder (ASD) is associated with disruption of local- and long-range functional connectivity (FC). The direction of those changes in FC (increase or decrease), however, is inconsistent across studies. Further, age-dependent changes of distance-specific FC in ASD remain unclear. In this study, we used resting-state functional magnetic resonance imaging data from sixty-four typical controls (TC) and sixty-four patients with ASD, whom we further classified into child (<11 years), adolescent (11–18 years) and adult cohorts (>18 years). Functional connectivity (FC) analysis was conducted at voxel level. We employed a three-way analysis of covariance on FC to conduct statistical analyses. Results revealed that patients with ASD had lower FC than TC in cerebellum, fusiform gyrus, inferior occipital gyrus and posterior inferior temporal gyrus. Significant diagnosis-by-distance interaction was observed in ASD patients with reduced short-range and long-range FC in posterior cingulate cortex and medial prefrontal cortex. Importantly, we found significant diagnosis-by-age-by-distance interaction in orbitofrontal cortex with short-range FC being lower in autistic children, but –to a less extent– higher in autistic adults. Our findings suggest a major role of connection length in development changes of FC in ASD. We hope our study will facilitate deeper understanding of the neural mechanisms underlying ASD.

Autism spectrum disorder (ASD) is a neurodevelopment disorder characterized by deficits in communication and social interaction, along with repetitive patterns of behavior and interests^1^. Neuroimaging studies demonstrated that ASD is associated with locally functional and structural deficits in the brain^2^,^3^. Nevertheless, the pathophysiology of autism remains largely unclear. Recently, the advent of functional connectivity (FC) analysis provided a new approach to understand the neural mechanism underlying ASD. FC analyses suggested that ASD is a disconnection syndrome, associated with alterations of connectivity between distributed brain areas rather than local deficits in specific brain regions^4^.

A number of previous studies indicated that patients with ASD have long-range under-connectivity and local over-connectivity^5^,^6^,^7^. However, not all evidence supports this hypothesis. Anderson and colleagues observed decreased inter-hemispheric FC in high-functioning autism, mainly in sensorimotor cortex, anterior insula and fusiform gyrus, indicating reduction of long-range connectivity^6^. By employing graph theory analysis, Keown and colleagues found increased local connectivity in adolescents with ASD in temporo-occipital regions^7^. They further found that the over-connectivity was associated with higher ASD symptom severity. Moreover, Courchesne and Pierce proposed that neuroinflammatory reactions such as glial activation, migration defects and excessive cerebral neurogenesis found in the frontal lobe are related to ASD^5^,^8^. According to the authors, these would be linked to local over-connectivity as well as long-distance fronto-cortical disconnection^9^. Other studies, however, reported findings that go against the hypothesis of long-range under-connectivity and local over-connectivity in ASD. For instance, decreased local FC in ASD was observed in the fusiform face area^10^ and in the somatosensory cortex^11^. Compromised short-distance fiber tracts in children with ASD were found in frontal, temporal and parietal lobes^12^. Furthermore, Supekar and colleagues reported hyper-connectivity across long-range and short-range connections in autistic children^13^. The inconsistent results highlighted above might be due to the heterogeneity of patients with ASD, the different age of cohorts recruited, as well as the different definition of ‘local’ and ‘long’ range FC. In consideration of the inconsistent findings reported in the literature, we posit that further studies of local- and long- FC in ASD across the lifespan are needed.

Development studies in the healthy brain revealed that short- and long-range FC differentially changes as a function of age. Specifically, it was demonstrated that long-range and short-range FC increases and decreases with age, respectively^14^,^15^,^16^. This developmental profile of FC in the human brain is thought to contribute to efficient information integration for higher cognition, as well as to segregation for specialized information processing. Given that ASD is a developmental disorder, we might speculate that this kind of development profile would become aberrant in this disease. To the best of our knowledge, there are only a few studies that investigated short- and long-range FC development in ASD. These revealed that short-range hyper-connectivity and long-range under-connectivity in autism may concurrently emerge due to problems with synapse pruning or formation^17^,^18^. Anderson and colleagues found a significant negative correlation of mean inter-hemispheric connectivity with age in healthy controls, which was absent in autism^6^. By employing regional homogeneity analyses, Dajani and Uddin observed disrupted short-range connectivity in ASD, with alterations mainly occurring in childhood rather than in adolescence and adulthood^19^. However, no study has systematically investigated distance-related development of FC in ASD yet.

Thus, the aim of the current study was to investigate the effect of anatomical distance on the development of FC in ASD. Currently, the data open sharing initiative of the Autism Brain Imaging Data Exchange (ABIDE) consortium provided us with a valuable opportunity to understand the mechanisms of development underlying ASD. The ABIDE aggregated and shared resting-state functional magnetic resonance imaging (R-fMRI) data sets from individuals with ASD and typical controls (TC). In consideration of the specific goal of the current study, we analyzed the data from ABIDE contributed by New York University Langone Medical Center, which included participants with age ranging from 6 years to 40 years. Based on previous findings, we hypothesized that patients with ASD showed abnormal development of FC for specific anatomical distances.

## Results

### Significant main effects and interaction effects

**Figure 1:**
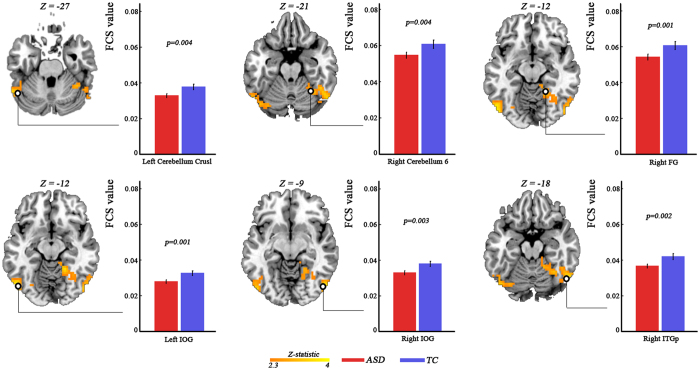
Significant main effect of diagnosis revealed by three-way analysis of covariance with frame-wise displacement as covariates. Statistical significance level was corrected for multiple comparisons using Gaussian random theory with *q* < 0.05 (*p* < 0.01, Z > 2.3). The bar plots indicate difference in FC between ASD and TC, as revealed by post-hoc analysis. FC, functional connectivity; ASD, autism spectrum disorder; TC, typically control; FG, fusifism gyrus; IOG, inferior occipital gyrus; ITGp, posterior inferior temporal gyrus.

**Figure 2:**
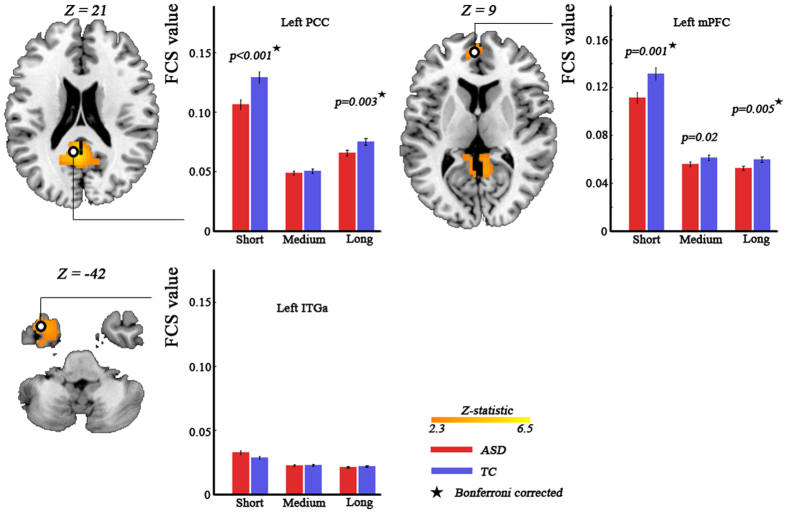
Significant diagnosis-by-distance interaction effect revealed by three-way analysis of covariance with frame-wise displacement as covariates Statistical significance level was corrected for multiple comparisons using Gaussian random theory with *q* < 0.05 (p < 0.01, Z > 2.3). The bar plots indicate difference in short-range, medium-range and long-range FC between ASD and TC. The stars donate bonferroni correction with *q* < 0.05. FC, functional connectivity; ASD, autism spectrum disorder; TC, typically control; PCC, posterior cingulate cortex; mPFC, medial prefrontal cortex; ITGa, anterior inferior temporal gyrus.

**Figure 3:**
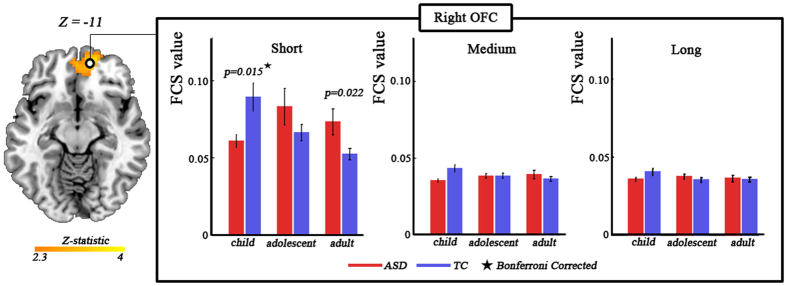
Significant diagnosis-by-age-by-distance interaction effect revealed by three-way analysis of covariance with frame-wise displacement as covariates. Statistical significance level was corrected for multiple comparisons using Gaussian random theory with *q* < 0.05 (*p* < 0.01, Z > 2.3). The bar plots indicate difference in short-range, medium-range, and long-range FC between ASD and TC at three age cohorts. The stars donate bonferroni correction with *q* < 0.05. FC, functional connectivity; ASD, autism spectrum disorder; TC, typically control; OFC, orbitofrontal cortex.

The analysis of covariance (ANCOVA) on FC values revealed a significant main effect of diagnosis (Fig. 1, Table 1), a diagnosis-by-distance interaction (Fig. 2, Table 1), and a diagnosis-by-age-by-distance interaction (Fig. 3, Table 1). Those significant effects were still observed not only at the voxel level but also using spherical regions of interests (ROIs) with 6 mm radius (Table S1). We did not observe a significant diagnosis-by-age interaction. Additionally, we found significant main effect of age, main effect of distance and age-by-distance interaction, which can be seen in Fig. S1. When applying correlation thresholds of 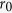 = 0.3 and 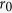 = 0.1 instead of 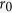 = 0.2, we observed similar results (Figs S2 and S3).

### Main effect of diagnosis

Significant main effect of diagnosis was observed mainly in right fusiform gyrus (partial η^2^ = 0.095; *F*_(1,365)_ = 12.65), left inferior occipital gyrus (partial η^2^ = 0.088; *F*_(1,365)_ = 11.64), right inferior occipital gyrus (partial η^2^ = 0.083; *F*_(1,365)_ = 10.90), right posterior inferior temporal gyrus ( = partial η^2^ 0.081; *F*_(1,365)_ = 10.65), left cerebellum crus1 (partial η^2^ = 0.068; *F*_(1,365)_ = 8.83) and right cerebellum 6 (partial η^2^ = 0.071; *F*_(1,365)_ = 9.19) (Fig. 1, Table 1). Post-hoc analysis revealed that FC of all those brain areas was lower in ASD patients as compared to TC (Fig. 1).

### Diagnosis-by-distance interaction

Remarkable diagnosis-by-distance interaction was found in bilateral posterior cingulate cortex (partial η^2^ = 0.117; *F*_(2,365)_ = 15.96), bilateral medial prefrontal cortex (partial η^2^ = 0.070; *F*_(2,365)_ = 9.15) and left anterior inferior temporal gyrus (partial η^2^ = 0.084; *F*_(2,365)_ = 11.03) (Fig. 2, Table 1). By employing post-hoc analysis, we found significant lower (Bonferroni corrected) short-range FC and long-range FC of both posterior cingulate cortex and medial prefrontal cortex in ASD patients as compared to TC (Fig. 2). In addition, there was a trend (*p* = 0.02, uncorrected) toward lower medium-range FC of medial prefrontal cortex (Fig. 2). We did not observe any group-difference in distance-based FC in left anterior inferior temporal gyrus between groups (Fig. 2).

### Diagnosis-by-age-by-distance interaction

We observed significant diagnosis-by-age-by-distance interaction in bilateral orbitofrontal cortex (partial η^2^ = 0.068; *F*_(4,365)_ = 4.44) (Fig. 3, Table 1). Post-hoc analysis revealed significant (Bonferroni corrected) diagnosis-by-age interaction on short-range FC (partial η^2^ = 0.078; *F*_(2,121)_ = 5.13; *p* = 0.007), but no significant interaction on medium-range FC (partial η^2^ = 0.054; *F*_(2,121)_ = 3.487; *p* = 0.034) and long-range FC (partial η^2^ = 0.029; *F*_(2,121)_ = 1.794; *p* = 0.171). We further found that the short-range FC was significantly lower (Bonferroni corrected) in autistic children, but a trend toward higher (*p* = 0.022, uncorrected) in autistic adults (Fig. 3).

## Discussion

In the current study, we investigated the influence of anatomical distance and age on FC changes in ASD. We found lowered FC in cerebellum, fusiform gyrus and occipital cortex in ASD. Moreover, patients with ASD showed reduced short- and long-range FC, but no difference in medium-range FC in posterior cingulate cortex and medial prefrontal cortex, suggesting distance-dependent dysfunction in ASD. Further, a significant diagnosis-by-age-by-distance interaction effect was observed in orbitofrontal cortex, suggesting a complex effect of age and anatomical distance on FC changes underlying ASD.

Firstly, we observed lowered FC of cerebellum in ASD. Abundant neuroimaging studies involved cerebellum in ASD. Motor tasks^20^,^21^ and language task^22^ abnormally activated specific cerebellum nucleus in ASD. Altered functional and anatomical FC of cerebellum in ASD was also observed^23^,^24^. Wang and colleagues stated that cerebellar damage is a strong risk factor for ASD, affecting a wide range of basic social capabilities^25^. Reduced FC of cerebellum found in the current study was in accordance with those studies, corroborating the idea that this brain structure has a crucial role in ASD. Additionally, we found relatively low FC in right posterior inferior temporal gyrus, fusiform gyrus, and bilateral inferior occipital cortex in patients with ASD. The posterior inferior temporal cortex was found to be involved in object perception^26^,^27^. Abnormal activation of right inferior temporal gyrus in autism during face discrimination task was observed, suggesting altered face recognition^27^. Face-processing deficits in ASD were also associated with dysfunction of fusiform area^28^ and inferior occipital cortex^29^, potentially as a consequence of neuronal loss within those brain areas^26^. Accordingly, we argue that the reduced FC of those brain areas observed in the current study may be related to abnormal face recognition in ASD.

A significant diagnosis-by-distance interaction was found in posterior cingulate cortex and medial prefrontal cortex. In both brain areas, patients with ASD showed reduced short- and long-range FC. A popular theory states that local over-connectivity and long-range under-connectivity are at the basis of ASD. For example, patients with ASD exhibited increased short-range FC within medial prefrontal cortex and posterior cingulate cortex^30^ and decreased long-range FC between the two brain areas^6^,^31^,^32^,^33^. However, some studies argued against that theory. For example, Supekar and colleagues found that children with ASD showed widespread hyper-connectivity not only at whole-brain and functional sub-systems levels, but also for both short- and long-range connections^13^. They further observed a relationship between hyper-connectivity and social deficits in children with ASD, suggesting specific mechanisms underlying this disorder during childhood. The age difference in the subject cohorts may contribute to inconsistency of the findings between studies. The lowered short- and long-range FC within the default mode network we observed in the current study support the under-connectivity hypothesis in patients with ASD. This is in line with a previous study^34^ reporting that patients with ASD had lowered local (short-range) as well as contralateral (long-range) FC of posterior cingulate cortex. Overall, the current results seem to suggest significant effect of anatomical distance on dysfunction of default mode network in ASD.

Interestingly, we observed a significant diagnosis-by-age-by-distance interaction in orbitofrontal cortex. The finding cannot just result from effects of motion displacement, as this was well-controlled in our study. Additionally, the finding was found to be robust with the use of different correlation thresholds (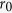 = 0.1, 0.2 and 0.3). The result implies that distance-dependent developmental dysfunction of orbitofrontal cortex may be involved in pathology of ASD. The orbitofrontal cortex plays key roles in social cognition and repetitive behaviors^35^, which are crucially involved in patients with ASD. Macrostructural deficits and functional connectivity impairments in the orbitofrontal cortex were previously associated with this disease^36^,^37^,^38^,^39^. In particular, a longitudinal study identified abnormal development of cortical thickness in lateral orbitofrontal cortex^39^. Our findings extend previous reports, stating that functional disconnections in ASD are not only related to age, but are associated with anatomical distance. Post-hoc analysis revealed that patients with ASD showed significantly diagnosis-by-age interaction in short-range FC, but no interaction in medium-range and long-range FC. This suggests that the developmental changes of FC in orbitofrontal cortex mainly occur within the brain regions itself. We further found that short-range FC of orbitofrontal cortex was lower in autistic children but higher in autistic adults, as compared to age-matched TC. The findings were in line with a previously structural study^36^, reporting smaller size of lateral orbitofrontal cortex in children with ASD, but larger size in adults with this disease. The potential mechanisms underlying the developmental impairment are still unclear, and future studies are warranted to address this question.

Several limitations of the current study should be considered. First, we used a definition of short-range, medium-range and long-range FC that was proposed in a previous network study^40^. The authors demonstrated that information communication along long-range connectivity travelled through rich-club areas and connections, communication along medium-range connectivity travelled through only rich-club regions, and that along short-range connectivity travelled mainly within local connections. This classification attributed different roles in brain communication to distinct distance-based FC groups. Nevertheless, this definition of short-range, medium-range and long-range FC is still arbitrary. Further studies are needed to systematically classify how alterations of FC in ASD exactly vary as a function of distance. Second, we investigated the abnormal development of FC by using a cross-sectional paradigm. Longitudinal studies are needed to confirm and better characterize developmental abnormalities in FC. Third, we investigated functional but not structural connectivity in ASD. It is well known that functional connectivity is largely constrained by anatomical pathways^41^. Accordingly, future studies are warranted to study the interaction between brain function and structure in ASD.

In conclusion, we found significant main effect of diagnosis with lower FC in cerebellum and face perception-related brain areas in ASD. These findings added to a growing literature demonstrating under-connectivity in ASD. In addition, we observed that patients with ASD showed distance-dependent decrease in FC within the default modal network. More importantly, we observed a significant diagnosis-by-age-by-distance interaction in the orbitofrontal cortex. Overall, these findings suggest that connection length plays a major role in development changes of FC in ASD. We hope our study will contribute to a deeper understanding of the neural mechanisms underlying ASD.

## Methods

### Participants

The data included in the current study were obtained from the ABIDE (http://fcon_1000.projects.nitrc.org/indi/abide/) database. Specifically, we used the data sets contributed by the New York University Langone Medical Center. Participants were classified into three age cohorts: children (<11 years, 20 TC and 18 ASD), adolescents (26 TC and 28 ASD, 11–18 years), and adults (>18 years, 18 TC and 18 ASD). The study was carried out in accordance with the Declaration of Helsinki. Experimental protocols were approved by the NYU institutional review board. Written informed consent was obtained from all participant. Participants with ASD were included if their deficits were classified as Autistic Disorder, Asperger’s Disorder, or Pervasive Developmental Disorder not otherwise specified based on the clinician’s DSM-IV-TR. In order to assess psychopathology for differential diagnosis and to determine comorbidity with Axis-I disorders, diagnostic assessments of ASD also included parent interview using the Schedule of Affective Disorders and Schizophrenia for Children-Present and Lifetime Version (KSADS-PL)^42^ for children (<17.9 years of age), and participant interview using the Structured Clinical Interview for DSM-IV-TR Axis-I Disorders, Non-patient Edition (SCID-I/NP) and the Adult ADHD Clinical Diagnostic Scale (ACDS)^43^ for adults (>18 years of age). Participants included in the TC group had no Axis-I disorder. For all participants, the exclusion criterion included current chronic systemic medical conditions, contraindications to MRI scanning, pregnancy and use of antipsychotics. The full IQ (FIQ), performance IQ (PIQ) and verbal IQ (VIQ) were estimated using the four subtests of the Wechsler Abbreviated Scale of Intelligence^44^. Age, gender, FIQ, PIQ, VIQ and head motion (characterized by frame-wise displacement [FD]) were well matched (p > 0.05) between TC and ASD within each age cohort. The details of demographic information can be found in Table 2.

### Data acquisition

All participants were scanned using a 3 Tesla SIEMENS MR scanner following diagnostic assessment. For the resting-state scan, subjects were asked to relax and look at a white cross-hair against a black background. Functional images were obtained a T2* MR sequence with using the following parameters: TR/TE = 2000/15 ms, 33 slices, 3 × 3 × 4 mm^3^ voxel size, 90^o^ flip angle, 240 mm FOV, 4 mm slice thickness, and 180 volumes in total. Anatomical images were acquired using a T1w MR sequence with the following parameters: TR/TE = 2530/3.25 ms, 1.3 × 1.0 × 1.3 mm^3^ voxel size, 7^o^ flip angle, 256 mm FOV, and 1.33 mm slice thickness.

### Data preprocessing

Data preprocessing was conducted using SPM8 software (http://www.fil.ion.ucl.ac.uk/spm/software/spm8/). Briefly, the first five time points were discarded to ensure magnetization stabilization. Functional images were then corrected for time-delays between slices, and for motion displacement between volumes. The mean FD across time points was calculated for each participant to characterize head motion. No participants had mean FD larger than 1 mm, so none of them was excluded from further analyses^45^,^46^. The functional MR images were spatially normalized to standard Montreal Neurological Institute (MNI) space using the unified segmentation tool of SPM8^47^ and then re-sampled to 3 × 3 × 3 mm^3^ voxel size. The normalization involved three steps: the anatomical image was first skull-stripped and co-registered to the corresponding functional image. The co-registered anatomical image was then segmented into gray matter, white matter, and cerebral spinal fluid (CSF), which generated normalization parameters donating transformation from native subject space to standard MNI space. The functional image was finally normalized into MNI space by using those normalization parameters.

Removal of covariates of no interests from the functional time series in MNI space was then performed by using multiple regression analysis. The covariates included 24-motion parameters and the first five top components obtained from the white matter mask and CSF mask by the using ‘CompCor’ method^48^. Previous studies demonstrated that the ‘CompCor’ method is more efficient compared to the mean-tissue signal method in removing the effect of cardiac and respiratory signals^48^,^49^. Considering that FC is sensitive to head motion^50^,^51^, especially in autism^52^, data scrubbing^50^ was conducted to reduce this negative effect. Specifically, ‘bad’ volumes characterized by FD larger than 1 mm^45^ were were identified and removed from the calculation of FC. Finally, the time series were then linear detrended and filtered at the range of 0.01–0.08 Hz.

### Functional connectivity analysis

Pearson correlation analysis was first performed between pairs of voxels for each subject within a mask defined by Automated Anatomical Labeling atlas^53^. This generated N × N correlation matrices, where N is the number of voxels (N = 54837 in our study). The Fisher r-to-z transformation was then applied to the correlation matrices to improve data normality. The z-score values in the transformed matrices represent FC between voxels. FC was classified into three groups bases on anatomical distance (L). Specifically, FC between a pair of voxel was considered short-range, medium-range or long-range FC if L was between 10 and 30 mm, between 30 and 90 mm and larger than 90 mm, respectively. This definition was in line with a previous study^40^. Of note, FC between voxels with L <= 10 mm was not included, which was to avoid spurious interpolation-driven connectivity induced by normalization^54^. For a given voxel 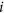, we computed its FC as follows:


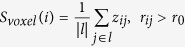


where 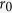 is a predefined threshold to eliminate weak correlations (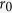 = 0.2 in the current study), 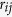 is the correlation value between voxel 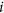 and voxel 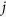, 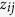 is the z-score obtained from 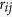 by using the Fisher transformation, 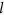 is a set of specific voxels, and 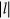 indicates the number of voxels within the set 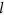. According to the formula above, long-distance FC of voxel 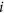 was calculated as the averaged z-scores across voxels whose L from voxel 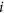 were larger than 90 mm. Likewise, we computed medium-distance FC and short-distance FC of voxel 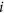 as above. By employing the procedures across all voxels, we obtained short-range, medium-range and long-range FC map for each participant. Those maps were finally smoothed at 6 mm full width half maximum. Notably, such a FC metric refers to “degree centrality” in the context of graph theory analyses. High FC is thought to indicate more efficient integration of information in the brain.

A three-way ANCOVA was conducted in SPM8 with diagnosis (two levels: ASD and TC) as group factor, age (three levels: child, adolescent and adult) as between-subject factor, distance (three levels: short, medium and long) as within-subject factor, and FD as covariate of no interest. Gaussian random field theory was employed to correct for multiple comparisons (*q* < 0.05, voxel *p* < 0.01, Z > 2.3) for each main effect and each interaction effect. To evaluate the effect of different correlation thresholds on our main results, we repeated FC computation and statistical analysis using other two correlation thresholds (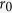 = 0.3 and 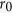 = 0.1, respectively).

Once any significant diagnosis-related effect was observed, we selected clusters from those statistical maps, thresholded using significance level corrected for multiple comparisons by Gaussian random theory, using the “pick cluster” function in XJView (http://www.alivelearn.net/xjview8/). For each cluster, the ROI was generated by selecting the spherical area with center at the peak and radius of 6 mm. In order to test whether there still were significant effects on the spherical ROIs, we performed another three-way ANCOVA analysis. Those ROIs were used for the following post-hoc analysis. For the main effect of diagnosis, we further investigated whether FC was higher or lower in ASD patients than TD. For the diagnosis-by-age interaction, we tested the group effect (ASD vs. TC) across the three age cohorts separately. Likewise, we examined group effect in short-range, medium-range and long-range FC for the diagnosis-by-distance interaction. For the ROIs showing significant diagnosis-by-age-by-distance interaction, we separately analyzed short-range FC, medium-range FC, and long-range FC of those ROIs using a two-way ANCOVA. If there were any significant effects in the diagnosis-by-age interaction, we further investigated the group effect across age cohorts. For each analysis step, we employed Bonferroni correction with *p* < 0.05/3 (three times in total at each step) to account for multiple comparisons.

## Additional Information

**How to cite this article**: Long, Z. *et al*. Alteration of functional connectivity in autism spectrum disorder: effect of age and anatomical distance. *Sci. Rep.* **6**, 26527; doi: 10.1038/srep26527 (2016).

## Supplementary Material

Supplementary Information

## Figures and Tables

**Table 1:** Main effect of diagnosis, diagnosis-by-distance interaction and diagnosis-by-age-distance interaction revealed by three-way ANCOVA analysis.

**Brain areas**	**L/R**	**Cluster Size**	**Peak coordinate**			**DOF**	**Partial η**^**2**^	***F*****-value**	***P*****-value**
			**x**	**y**	**z**				
Main effect of diagnosis									
Fusiform gyrus	R	88	21	−48	−12	1	0.095	12.65	0.001
Inferior occipital gyrus	L	62	−51	−75	−12	1	0.088	11.64	0.001
Inferior occipital gyrus	R	41	51	−75	−9	1	0.083	10.90	0.001
Posterior inferior temporal gyrus	R	98	54	−63	−18	1	0.081	10.65	0.001
Cerebellum crusl	L	69	−57	−57	−27	1	0.068	8.83	0.004
Cerebellum 6	R	42	33	−54	−21	1	0.071	9.19	0.003
Diagnosis-by-distance interaction									
Posterior cingulate cortex	L/R	212	−3	−51	21	2	0.117	15.96	<0.001
Medial prefrontal cortex	L/R	62	−3	54	9	2	0.070	9.15	<0.001
Anterior inferior temporal gyrus	L	81	−39	12	−42	2	0.084	11.03	<0.001
Diagnosis-by-age-by-distance interaction									
Orbitofrontal gyrus	L/R	188	15	54	−12	4	0.068	4.44	0.002

Statistical significance level is corrected for multiple comparisons using Gaussian random theory with q < 0.05 (voxel p < 0.01, Z > 2.3). The peak coordinate is defined in MNI space. ANCOVA, analysis of covariance; L, left; R, right; DOF, degree of freedom.

**Table 2:** Demographic and clinical information for ASD group and TC group.

**Demographic data**	**Child cohort**			**Adolescent cohort**			**Adult cohort**		
	**ASD**	**TC**	**P**	**ASD**	**TC**	**P**	**ASD**	**TC**	**P**
Gender (M/F)	17/1	19/1	0.94^a^	23/5	21/5	0.90^a^	14/4	14/4	1^a^
Age (years) (mean ± sd)	9.6 ± 1.0	9.3 ± 1.5	0.44^b^	13.7 ± 1.8	14.5 ± 1.9	0.14^b^	25.4 ± 5.9	25.5 ± 4.2	0.97^b^
FIQ (mean ± sd)	110.6 ± 20.0	112.2 ± 12.3	0.77^b^	103.6 ± 13.5	104.3 ± 13.5	0.85^b^	108.1 ± 13.9	110.1 ± 7.9	0.59^b^
Mean FD (mm) (mean ± sd)	0.14 ± 0.05	0.13 ± 0.05	0.43^b^	0.16 ± 0.08	0.13 ± 0.07	0.24^b^	0.11 ± 0.04	0.10 ± 0.04	0.71^b^

ASD, autism spectrum disorder; TC, typical controls; FIQ, full IQ; P, p-value; M, male; F, female; FD, frame-wise displacement.

^a^Statistical significance level was calculated using a chi-square test.

^b^Statistical significance level was computed using a two-tailed two-sample t-test.
