# Supplementary material for: Alteration of functional connectivity in autism spectrum disorder: effect of age and anatomical distance
Source: Sci Rep. 2016 May 19;6:26527. doi: 10.1038/srep26527 (PMC4872225; doi:10.1038/srep26527)
Supplement: Supplementary Information [file srep26527-s1.doc]

**Supplementary information of:**

**Alteration of functional connectivity in autism spectrum disorder: effect of age and anatomical distance**

**Zhiliang Long ^a^; Xujun Duan ^a^; Dante Mantini ^b,c,d^,*; Huafu Chen ^a^,***

^a^ Center for Information in BioMedicine, Key laboratory for Neuroinformation of Ministry of Education, School of Life Science and Technology, University of Electronic Science and Technology of China, Chengdu 610054, China.

^b^ Neural Control of Movement Laboratory, ETH Zurich, Switzerland.

^c^ Department of Experimental Psychology, University of Oxford, Oxford, United Kingdom.

^d^ Laboratory of Movement of Control and Neuroplasticity, KU Leuven, Leuven, Belgium.

* Corresponding author:

Huafu Chen, Center for Information in BioMedicine, Key laboratory for Neuroinformation of Ministry of Education, School of Life Science and Technology, University of Electronic Science and Technology of China, Chengdu 610054, China. E-mail: chenhf@uestc.edu.cn (H. Chen) and Dante Mantini, Laboratory of Movement of Control and Neuroplasticity, KU Leuven, Leuven, Belgium. Email: dante.mantini@kuleuven.be

**Supplementary Table S1. Statistical analysis of main effect of diagnosis, diagnosis-by-distance interaction and diagnosis-by-age-by-distance interaction using regions of interests with 6 mm radius.**

| Regions of interests | Partial η^2^ | *F*-value | *P*-value |
| --- | --- | --- | --- |
|
| ***Main effect of diagnosis*** | | | |
| Right fusiform gyrus | 0.081 | 10.79 | 0.001 |
| Left inferior occipital gyrus | 0.09 | 12.18 | 0.001 |
| Right inferior occipital gyrus | 0.069 | 9.11 | 0.003 |
| Right posterior inferior temporal gyrus | 0.075 | 9.99 | 0.002 |
| Left cerebellum crusl | 0.066 | 8.72 | 0.004 |
| Right cerebellum 6 | 0.066 | 8.66 | 0.004 |
| ***Diagnosis-by-distance interaction*** | | | |
| Left posterior cingulate cortex | 0.101 | 13.8 | <0.001 |
| Left medial prefrontal cortex | 0.057 | 7.36 | 0.001 |
| Left anterior inferior temporal gyrus | 0.09 | 11.98 | <0.001 |
| ***Diagnosis-by-age-by-distance interaction*** | | | |
| Right orbitofrontal gyrus | 0.071 | 4.60 | 0.001 |


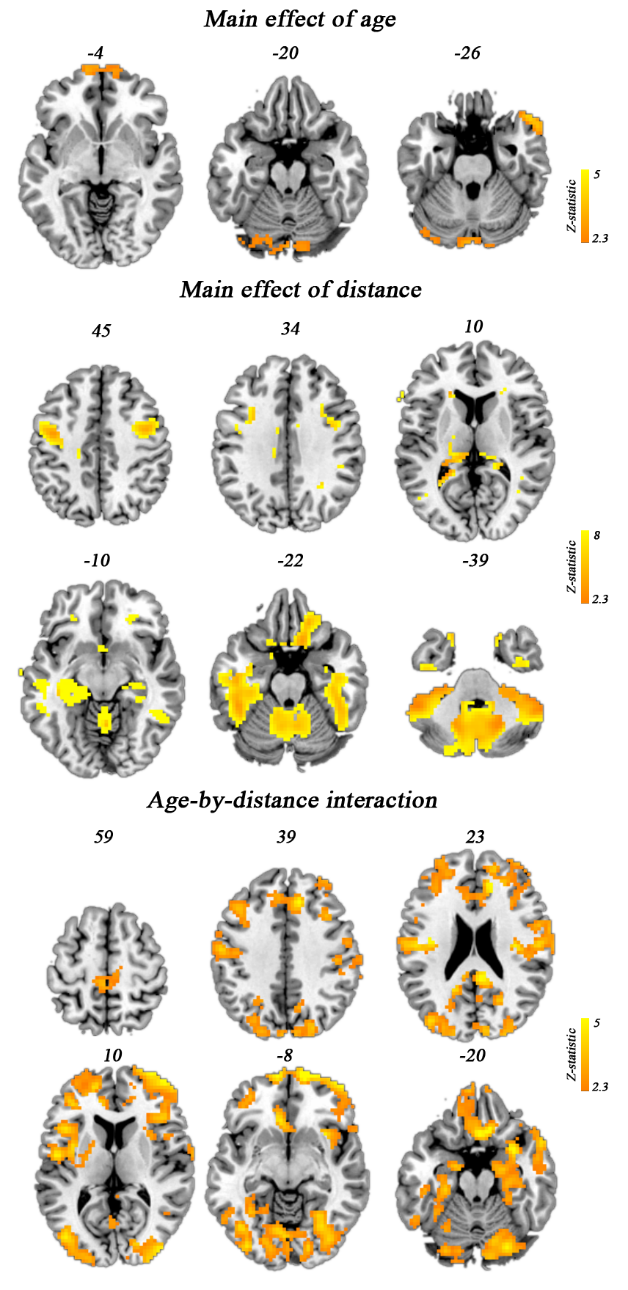


**Supplementary Figure S1.** Significant main effect of age, main effect of distance and age-by-distance interaction revealed by three-way analysis of covariance with frame-wise displacement as covariates. Of note, the results were obtained using a correlation threshold of 0.2.


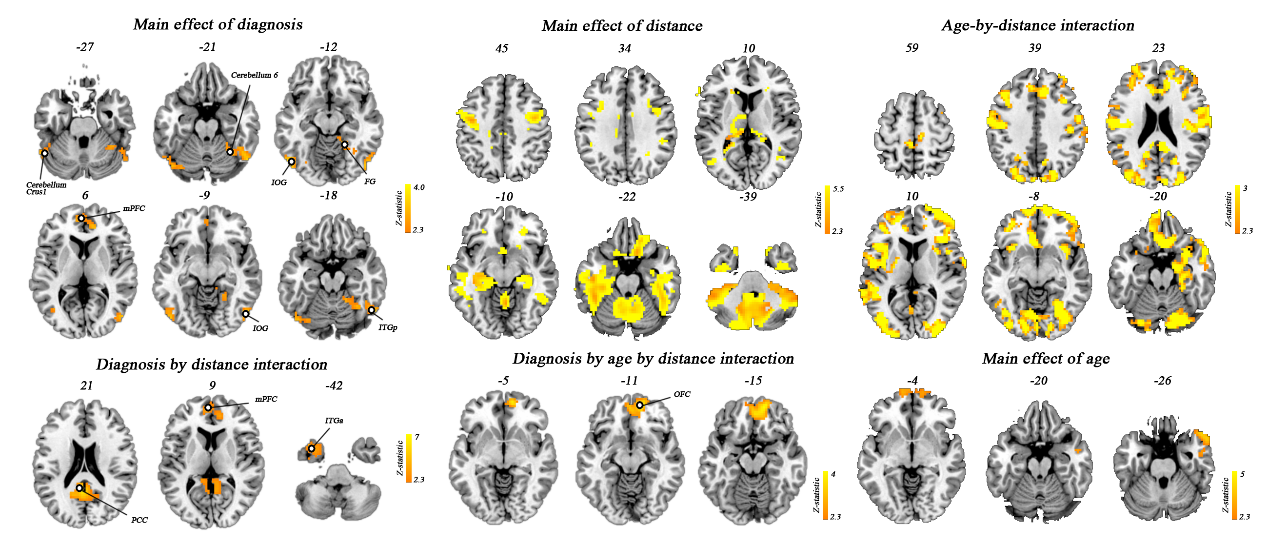


**Supplementary Figure S2.** Significant main effect of diagnosis, main effect of age, main effect of distance, diagnosis-by-distance interaction, age-by-distance interaction and diagnosis-by-age-by-distance interaction revealed by three-way analysis of covariance with frame-wise displacement as covariate. Of note, the results were obtained using a correlation threshold of 0.3. FG, fusifism gyrus; IOG, inferior occipital gyrus; ITGp, posterior inferior temporal gyrus. PCC, posterior cingulate cortex; mPFC, medial prefrontal cortex; ITGa, anterior inferior temporal gyrus.


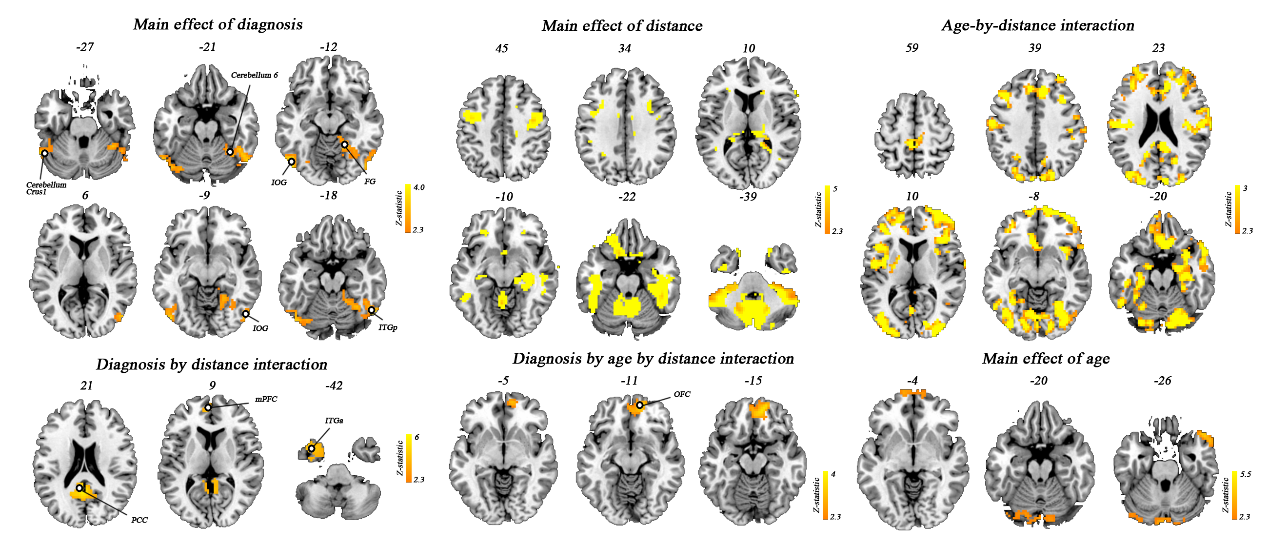


**Supplementary Figure S3.** Significant main effect of diagnosis, main effect of age, main effect of distance, diagnosis-by-distance interaction, age-by-distance interaction and diagnosis-by-age-by-distance interaction revealed by three-way analysis of covariance with frame-wise displacement as covariate. Of note, the results were obtained using a correlation threshold of 0.1. FG, fusifism gyrus; IOG, inferior occipital gyrus; ITGp, posterior inferior temporal gyrus. PCC, posterior cingulate cortex; mPFC, medial prefrontal cortex; ITGa, anterior inferior temporal gyrus.
